# Supplementary material for: Prevalence and factors associated with underweight among children under five born to adolescent mothers: Evidence from the 2022 Kenya Demographic and Health Survey
Source: PLoS One. 2026 Feb 10;21(2):e0342460. doi: 10.1371/journal.pone.0342460 (PMC12890162; doi:10.1371/journal.pone.0342460)
Supplement: Attachment — Submitted filename: Response to Reviewers.pdf [file pone.0342460.s003.pdf]

POINT-BY-POINT RESPONSE TO THE REVIEWERS' COMMENTS

Ref: PONE-D-25-46014

Factors associated with underweight among children under five born to adolescent mothers: Evidence from the 2022 Kenya Demographic and Health Survey

Dear Editor and Reviewer,

We sincerely thank the Editor and Reviewer #2 for their careful evaluation of our manuscript entitled "Prevalence and factors associated with underweight among children under five born to adolescent mothers: Evidence from the 2022 Kenya Demographic and Health Survey." We appreciate the constructive comments, which have helped to improve the clarity, methodological transparency, and overall quality of the manuscript.

Below, we provide a point-by-point response to each comment raised by Reviewer #2. All revisions have been incorporated into the revised manuscript, with changes highlighted in the tracked-changes version.

Reviewer #2 – Comment 1

"*Though the author has newly added a flow chart, which is good for sample selection and exclusion criteria, it is not well-defined. Please use 'Yes', 'No' and directional signs in the proper place from 1st to the last step*."

Response:

We thank the reviewer for this helpful suggestion. The flowchart has been revised to improve clarity and logical flow. Specifically, we have:

- Reorganised the sequence from the first to the final step of sample selection,
- Added clear directional arrows to guide the reader,
- Explicitly indicated inclusion and exclusion steps using consistent labels and directional flow.

Reviewer #2 – Comment 2

"*In table 1, it mentions 'Characteristics of the study participants (Unweighted N=819)' but in the table header uses weighted frequency. Please check it carefully*."

Response:

Thank you for highlighting this inconsistency. We have carefully reviewed Table 1 and confirm that the frequencies presented are weighted, in line with DHS analytical recommendations. The table title has now been corrected to indicate a weighted sample size (Weighted N = 819) to ensure consistency between the table heading and the reported statistics.

Reviewer #2 – Comment 3

"*I wonder how the author could calculate the VIF value for a categorical variable and why they described 'as high values indicate multicollinearity and unstable coefficients' instead of the standard cutoff value of VIF. The author should acknowledge this issue in the limitations section."*

Response:

We thank the reviewer for this important methodological observation. We have addressed this concern in two ways:

- Specified standard cutoff values for VIF interpretation (VIF > 5 indicating moderate multicollinearity and VIF > 10 indicating serious multicollinearity).
- Explicitly acknowledged the limitation of applying VIF in models including categorical predictors and non-linear regression in the Strengths and Limitations section, noting that residual multicollinearity may not be fully captured and that coefficient estimates should be interpreted with caution.
