# Supplementary material for: Prevalence and factors associated with underweight among children under five born to adolescent mothers: Evidence from the 2022 Kenya Demographic and Health Survey
Source: PLoS One. 2026 Feb 10;21(2):e0342460. doi: 10.1371/journal.pone.0342460 (PMC12890162; doi:10.1371/journal.pone.0342460)
Supplement: Attachment — Submitted filename: Response to Reviewers.docx [file pone.0342460.s002.docx]

**POINT-BY-POINT RESPONSE TO THE REVIEWERS’ COMMENTS**

**Ref: PONE-D-25-46014**

Factors associated with underweight among children under five born to adolescent mothers in Kenya: Evidence from the 2022 Kenya Demographic and Health Survey

**REVIEWER #1:**

**GENERAL COMMENTS:** We sincerely thank Reviewer 1 for their constructive comments and positive evaluation. We have carefully proofread the manuscript to correct the minor typographical issues noted. These included standardizing decimal places in percentages and ensuring consistency in formatting across tables and references.

**REVIEWER #2:**

**COMMENT 1:** In the abstract, the author mentioned, “This study examined the prevalence and factors associated with underweight”. I think the author should add “prevalence” in the title.

**Response:** Thank you for this suggestion. We agree with your recommendation and have revised the title accordingly. **Revised Title:** *Prevalence and factors associated with underweight among children under five born to adolescent mothers in Kenya: Evidence from the 2022 Kenya Demographic and Health Survey*

**COMMENT 2:** The method section will be more understandable if the author creates a short flow chart for study design and data processing, including the inclusion and exclusion criteria, the number of weighted and unweighted observations, and missing values.

**Response:** Thank you for the helpful suggestion. In response, we have developed and included a flowchart (Figure 1) in the methods section summarising study design, inclusion/exclusion criteria, and sample derivation.

**COMMENT 3:** Use a reference for the VIF justification and explain the meaning of VIF.

**Response:** We retained the sentence and expanded the explanation with a supporting citation:

**Revised Section in Methods:** "Multicollinearity was assessed using the variance inflation factor (VIF) to determine the extent of the correlation among the independent variables. The VIF helps identify predictors that may distort regression estimates, as high values indicate multicollinearity and unstable coefficients (O’Brien, 2007)."

**COMMENT 4:** Justify why a p-value threshold of 0.2 was used for including variables in multivariable analysis.

**Response:** The methods section now states: "A bivariate model was first fitted, and independent variables with a p-value less than 0.20 were included in the multivariable analysis to avoid excluding potentially important predictors. This threshold allows the inclusion of variables that may not reach conventional levels of significance in the bivariate analysis but could be significant in the multivariable context due to confounding or interaction (Hosmer et al., 2013)."

**COMMENT 5:** Fix inconsistent use of decimal places in percentages.

**Response:** All percentages in text and tables have been standardized to two decimal place.

**COMMENT 6:** N value inconsistencies in Table 2.

**Response:** These discrepancies have been corrected.

**COMMENT 7:** Reference categories missing in regression tables.

**Response:** Reference categories are now explicitly labelled in all relevant tables.

**COMMENT 8:** Confirm whether sampling weights were used in modelling.

**Response:** The methods section has been updated to confirm that all estimates, including regression models, accounted for survey design using the "svy" command in Stata.

**COMMENT 9: Redundancy between Tables 3 and 4.**

**Response:** We merged the bivariate and multivariable results into a single comprehensive table to avoid redundancy. This change is reflected in the Results section.

**COMMENT 10:** Of all the independent variables there shows only two variables are significant; justification needed. Also, bivariate chi-square test showed 80% non-significance.

**Response:** Thank you for your observation. We provide the following justification regarding our model-building strategy and interpretation of results:

Many variables did not show statistical significance in the initial bivariate analysis, as presented in Tables 2 and 3 (CPR column). This is why we used a more inclusive p-value threshold of 0.20 for variable selection in the multivariable model, a common epidemiological practice to avoid excluding important confounders. Additionally, we retained key variables known from prior literature on undernutrition regardless of their bivariate significance to ensure a well-adjusted model. The multivariable analysis aims to identify independent predictors by adjusting for confounders. It is expected that some variables significant in bivariate analysis become non-significant after adjustment. For example, maternal secondary education was significant in the crude analysis but not in the adjusted model, suggesting its effect is mediated by other factors. Therefore, the finding that only marital status and child’s age remain significant demonstrates these are the strongest independent predictors of underweight, reinforcing rather than weakening our conclusions. This approach ensures robust identification of key factors affecting underweight among children of adolescent mothers.

**REVIEWER #3:**

**COMMENT 1:** Shift reference (5) to the end of the sentence.

**Response:** This has been corrected in the Introduction section.

**COMMENT 2: Citation style inconsistency.**

**Response:** All in-text citations and references were revised to comply with the Vancouver style used by PLOS ONE. We used EndNote Referencing software to ensure consistency.

**COMMENT 3: Add mention of management guidelines for underweight.**

**Response: Response:** Thank you for this valuable suggestion. We agree that linking our study's implications to established guidelines is crucial.

We have revised the discussion section to explicitly state that our recommendations are directly informed by and aligned with existing public health and nutritional guidelines for managing undernutrition. This clarification strengthens the basis for our proposed interventions.

The revised text now reads: "These findings highlight the need for public health interventions to support adolescent mothers in adopting appropriate complementary feeding practices... In line with established public health and nutritional guidelines, interventions should prioritise enhancing maternal knowledge, promoting dietary diversity, and improving access to nutritionally adequate foods for their children.

**COMMENT 4:** Expand explanation of sampling procedure.

**Response:** We added details on KDHS's two-stage stratified sampling method in the Methods section.

**Comment 5:** Discuss feeding practices.

**Response**: We acknowledged that the dataset lacked detailed feeding data for this subgroup and stated this limitation section of the manuscript.

**COMMENT 6:** Clarify wealth index construction.

**Response:** We noted that the wealth index was derived using PCA, following DHS standard methodology.

**COMMENT 7:** Clarify marital status definitions.

**Response:** Thank you for your comment. Our study used secondary data from the 2022 Kenya Demographic and Health Survey, where marital status was classified into standard categories as follows: not married (including single or never married), married (currently in a legal marital union), cohabiting (living together without formal marriage), and previously married (divorced, separated, or widowed).

**COMMENT 8:** Present results as percentages.

**Response:** The Results section was revised to emphasise percentage reporting over absolute counts.

**COMMENT 9:** Use dash instead of comma for CI.

**Response:** Fixed throughout manuscript (e.g., 95% CI: 8.65% - 13.97%).

**COMMENT 10:** The sentence “sub-Saharan Africa continues to report over 100 births per 1,000 adolescent girls, with approximately 6.1 million births annually among those aged 15 to 19 years (10)” was duplicated in the Discussion.

**Response:** Thank you for this observation. The repeated sentence has been removed from the Discussion section to avoid redundancy. The original statement remains appropriately cited in the Introduction.
